# Pathogenic *Leptospira* spp. in Bats, Madagascar and Union of the Comoros

**DOI:** 10.3201/eid1810.111898

**Published:** 2012-10

**Authors:** Erwan Lagadec, Yann Gomard, Vanina Guernier, Muriel Dietrich, Hervé Pascalis, Sarah Temmam, Beza Ramasindrazana, Steven M. Goodman, Pablo Tortosa, Koussay Dellagi

**Affiliations:** Centre de Recherche et de Veille sur les Maladies Émergentes dans l'Océan Indien, St. Clotilde, La Réunion, France (E. Lagadec, Y. Gomard, V. Guernier, M. Dietrich, H. Pascalis, S. Temmam, P. Tortosa, K. Dellagi);; Université de Lyon, Villeurbanne, France (E. Lagadec, S. Temmam);; Université de La Réunion, St. Clotilde (M. Dietrich, P. Tortosa);; Université d’Antananarivo, Antananarivo, Madagascar (B. Ramasindrazana);; Association Vahatra, Antananarivo (B. Ramasindrazana, S.M. Goodman);; Field Museum of Natural History, Chicago, Illinois, USA (S.M. Goodman);; and Institut de Recherche pour le Développement, St. Clotilde (Y. Gomard, V. Guernier, H. Pascalis, S. Temmam, K. Dellagi)

**Keywords:** Leptospira, bats, southwestern Indian Ocean islands, endemic, Union of the Comoros, Madagascar, bacteria, zoonoses

**To the Editor**: Leptospirosis is a zoonosis of global distribution; incidence rates are particularly high in tropical areas (*1*). Leptospirosis is a major public health problem on islands in the southwestern Indian Ocean, particularly La Réunion, Mayotte, and the Seychelles (where incidence rates are among the highest in the world) (*1*). In contrast, no human case has been reported on the nearby islands of Madagascar and Union of the Comoros. However, the recent demonstration of pathogenic *Leptospira* spp. in small mammals introduced to Madagascar suggests possible transmission from free-living animals to humans (*2*).

In addition to the fact that incidence rates vary among humans, clinical bacterial isolates from different islands belong to different serogroups and serovars and show diverse molecular features (*3*,*4*). This diversity might be correlated with that of the reservoir hosts; the islands in the southwestern Indian Ocean are a hot spot of biodiversity with extraordinary levels of vertebrate endemism. Most studies investigating wild-animal reservoirs of *Leptospira* spp. on the islands in the southwestern Indian Ocean have focused on small mammals that had been introduced to the islands (*2*,*5*), although bats infected with pathogenic *Leptospira* spp. have been identified in other regions (*6*). Whether bats are a reservoir of *Leptospira* spp. on these islands remains unknown. Therefore, we looked for this bacterium in bats from Madagascar and Union of the Comoros and characterized associated genetic diversity.

As part of an ongoing program aimed at identifying viral and bacterial infectious agents in island wild fauna, 129 insectivorous and frugivorous bats were tested for *Leptospira* spp. The bats belonged to 12 species: 9 from Madagascar (*Mormopterus jugularis*, *Otomops madagascariensis*, *Triaenops furculus*, *T*. *menamena*, *Miniopterus gleni*, *Miniopterus griffithsi*, *Miniopterus mahafaliensis*, *Myotis goudoti*, *Hypsugo anchietae*) and 3 from Union of the Comoros (*Rousettus obliviosus*, *Chaerephon pusillus*, *Miniopterus griveaudi*). Bats were captured in mist nets or harp traps at 8 sites in Madagascar and 6 sites in Union of the Comoros. Organs were collected in the field and immediately stored in liquid nitrogen.

Total nucleic acids were extracted from a pool of kidney, spleen, and lung tissue by using the Biorobot EZ1 and EZI Virus Mini Kit version 2.0 (QIAGEN, Les Ulis, France). Reverse transcription was then performed with GoScript reverse transcriptase (Promega, Charbonnières-les-Bains, France) to obtain cDNA. We screened pathogenic *Leptospira* spp. with a probe-specific real-time PCR (*7*). The 25 positive samples were subsequently subjected to a PCR procedure that amplified fragments from 682 to 1,293 bp of the 16S rRNA gene (depending on the amplification success) by using published primers (*8*–*10*). Resulting PCR products from 7 samples were sequenced and compared with available sequences in GenBank by using phylogenetic construction with PhyML 3.0.

Of the 12 bat species tested, 11 were positive for *Leptospira* spp. (the only *H. anchietae* bat tested was negative). Among 52 bats from Madagascar, 18 (34.6%) were infected; detection rates were often high, e.g., 8 (80%) of 10 *T. menamena* bats. In contrast, among 77 bats from Union of the Comoros, only 9 (11.7%) were infected. *Leptospira* spp. seem to be ubiquitous in the study areas; infected bats were found at 7 of 8 sites in Madagascar and 3 of 6 sites in the Union of the Comoros. Of the 7 *Leptospira* spp. sequences obtained from bats in this study, 3 were closely related to *L*. *borgpetersenii*, 1 grouped with *L*. *interrogans*, and 3 were not associated with any described species (Figure). *L*. *interrogans* and *L*. *borgpetersenii* were identified from *R*. *obliviosus* bats from the same cave in the Union of the Comoros, and the *L*. *borgpetersenii* sequence was closely related to that identified from the *O. madagascariensis* bats, which are endemic to Madagascar. Potentially pathogenic *Leptospira* spp. were found in bats of a wide variety of species in Madagascar and Union of the Comoros, at most study sites, and at levels notably higher than those reported from similar studies in other regions (*6*). Some of the bats that were *Leptospira* spp.–positive by PCR, particularly the genera *Mormopterus* and *Chaerephon*, often occupy synanthropic day roost sites. For example, we sampled 1 positive colony of *C*. *pusillus* bats in a school attic (Pomoni, Anjouan, Union of the Comoros), and bat scats were visible on the floor within the classroom.

**Figure:**
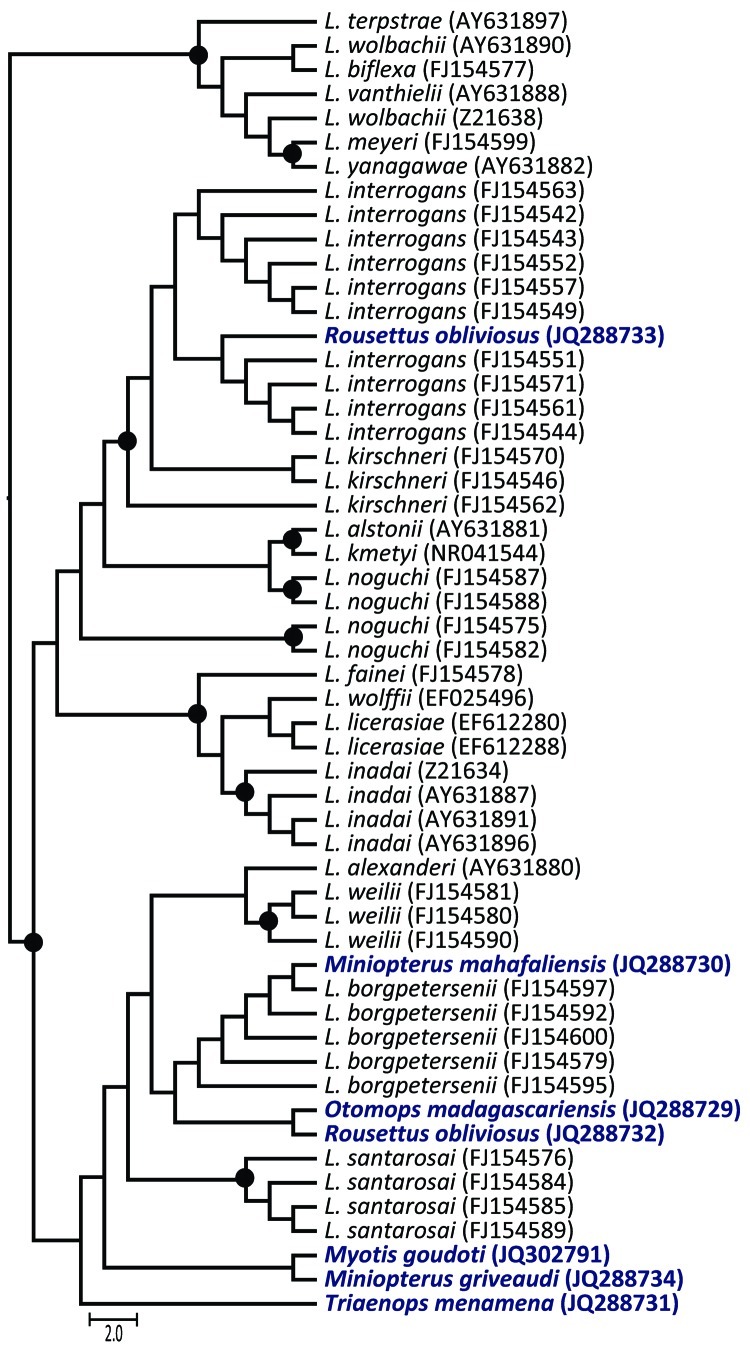
Maximum-likelihood phylogenetic tree of *Leptospira* spp.16s rDNA in bats from Madagascar and the Union of the Comoros. The dendrogram was constructed with a fragment of 641 bp, with the TIMef+I+G substitution model, and with 1,000 replications. Only bootstrap supports >70% are shown (circles). The precise geographic information of the sampled bats can be accessed through the GenBank accession numbers indicated in parentheses at branch tips. Host bat species for the sequences generated in this study are shown in **boldface**. Scale bar indicates number of nucleotide substitutions per site.

Bats from Madagascar and Union of the Comoros harbor a notable diversity of *Leptospira* spp.; this finding is in accordance with the diversity found in a comparable investigation of bats in the Amazon region (*6*). Although leptospirosis in humans is suspected only on the islands associated with this study (*10*), incidence among humans in Mayotte, part of the Union of the Comoros archipelago, has been shown to be high and mainly associated with *L*. *borgpetersenii* (*3*). The use of more polymorphic markers combined with the sequencing of clinical isolates should provide better characterization of *Leptospira* spp. diversity and the potential role of bats in human leptospirosis.
